# Perceptions of Psychological Coercion and Human Trafficking in the West Midlands of England: Beginning to Know the Unknown

**DOI:** 10.1371/journal.pone.0153263

**Published:** 2016-05-05

**Authors:** Coral J. Dando, David Walsh, Robin Brierley

**Affiliations:** 1 University of Wolverhampton, Institute of Psychology, Wolverhampton, United Kingdom; 2 Derby University, Dept. of Law and Criminology, Derby, United Kingdom; Örebro University, SWEDEN

## Abstract

Modern slavery is less overt than historical state-sanctioned slavery because psychological abuse is typically used to recruit and then control victims. The recent UK Draft Modern Slavery Bill, and current UK government anti-slavery strategy relies heavily on a shared understanding and public cooperation to tackle this crime. Yet, UK research investigating public understanding of modern slavery is elusive. We report community survey data from 682 residents of the Midlands of England, where modern slavery is known to occur, concerning their understanding of nonphysical coercion and human trafficking (one particular form of modern slavery). Analysis of quantitative data and themed categorization of qualitative data revealed a mismatch between theoretical frameworks and understanding of psychological coercion, and misconceptions concerning the nature of human trafficking. Many respondents did not understand psychological coercion, believed that human trafficking did not affect them, and confused trafficking with immigration. The public are one of the most influential interest groups, but only if well informed and motivated towards positive action. Our findings suggest the need for strategically targeted public knowledge exchange concerning this crime.

## Introduction

Modern day slavery is akin to the state-sanctioned chattel slavery common in imperial Rome [1] and America in the 1600s [2]. However, in contrast to historical slavery systems characterized by whips, chains, and physical imprisonment, modern day slavery is less overt, typically with no obvious visible signs of restraint. Rather, psychological abuse, coercion and mental manipulation play a powerful role in forcing modern day slaves to work in a variety of industries [3, 4]. Psychological abuse and coercion are easier to conceal than more physical forms of restraint and control and so modern slavery represents significant challenges in terms of both recognition and prevention.

Over the past few years, prompted in part by the draft Modern Slavery bill [5], there have been a number of high profile calls for the UK public to ‘open their eyes and ears’ and assist the authorities in combating one form of modern slavery, namely human trafficking [6–8]Human trafficking involves the illegal trade of men, women and children into conditions of exploitation for commercial gain, using deception, psychological coercion, the abuse of power, and/or the abuse of vulnerability [9]. Trafficking has received an increasing amount of national and international attention over the past decade. However, recognising, reporting, preventing, and collecting data on human trafficking remains stubbornly challenging, and so precise trafficking statistics remain elusive. Evidence *that* human trafficking occurs, and its impact on both victims and society are widely reported and generally acknowledged, worldwide [10]. There is a consensus that tens of thousands of people become victims each year, that trafficking is a very profitable form of transnational crime [11–14]and that the numbers of people being trafficked is increasing and are expected to continue to grow [9, 15, 16]

The UK government’s Modern Slavery Strategy [5] aims to significantly reduce the prevalence of human trafficking by, among other things, improving awareness of the signs of human trafficking amongst the general public (p. 10). Hence, the general public is a key constituent in tackling this crime.

There numerous websites providing information about the existence and signs of human trafficking *per se*, how to report suspected cases, and where/how victims can access information and assistance. However, most fail to highlight, and in some cases even acknowledge the role of psychological abuse and coercion: what it is, how to spot the subtle signs, and how it is used to target and control victims (e.g. National Crime Agency; Police Scotland; Crimestoppers; indirect etc.). Hence, public understanding of psychological abuse and coercion, and the role this type of mental manipulation plays in recruiting and controlling victims of human trafficking may be limited.

Understanding public perceptions is important because perceptions directly influence behaviour in a top down manner, as consistently demonstrated by researchers, worldwide. For example, perceptions of risk and vaccine uptake [17] need and international global aid contributions [18] cultural distance and anti-immigration policy [19] and the efficacy of CCTV cameras, and public acceptability [20] Public awareness/understanding that lags behind reality can have a negative impact on governmental aspirations and outcomes. If perceptions and/or understanding of human trafficking and psychological coercion are misguided, then despite best intentions the public cannot support the authorities because they do not know what to open their ears and eyes too. Equally, if understanding of trafficking, its consequences and impact for the victims and society are not fully understood it may be that the public simply closes their eyes and ears, even if/when they do experience, or suspect this crime.

The only available (to our knowledge) UK data reports the following headline results [21], i) 24% of respondents did not know what human trafficking was, ii) 34% believed that human trafficking was related to immigration, and iii) almost 20% associated human trafficking with prostitution, only. Similar findings are reported from other countries (e.g., Russia, Ukraine, & Hungary: [21, 22]). However, this research did not investigate perceptions/knowledge of psychological coercion, and geographical sample details are not reported. The research reported here represents a significant first step toward understanding perceptions/understanding of human trafficking and psychological coercion in one area of the West Midlands of England where the trafficking of children and adults is known to occur [9, 23]. Indeed, the number of referrals of trafficked victims by first responders in the West Midlands is higher than the national average [9], and it is here that the first multi agency anti-trafficking network was formed in 2008 in response to an increasing need to support a greater understanding of the rapidly changing realities of human trafficking, as witnessed by those working on the ground. Given this, the aims of this research were to investigate *what* residents of this area of England understand human trafficking and psychological coercion to be, and the source of their knowledge.

### Psychological Coercion

The existence and impact of psychologically coercive methods of constraining and manipulating individuals has long been acknowledged, for example, in international human rights rulings and policies, national and international legislation [24, 25] and the Geneva Convention on prisoners of war [26].

Psychological coercion is known to be a dominant feature of human trafficking [5, 27]. A recent self-report study of the experiences of trafficked victims [28] found much of Biderman’s framework of coercion [29] present in victims’ accounts–isolation, increased dependency upon the abuser, introspection, perception distortion, and disequilibrium (brought about by the abuser alternating between charm and seduction and aggressive threats). US agency officials involved in the investigation of human trafficking (or associated with supporting victims) have also reported analogous findings [4].

Biderman’s framework of psychological coercion [29], which came into being some 50 years ago, but has only recently come to the fore following reports that his principles had been used against suspected terrorists in Guantanamo Bay [28, 30], was first constructed in the context of interrogative torture and it involves a number of facets: i) deprivation of social support and contact, increased dependency, and forced introspection; ii) aggressive credible threats if the victim’s behaviour is not compliant, which creates anxiety and despair; iii) humiliation and degradation to induce psychological and physical exhaustion; iv) demonstrations of power by the use of trivial, contradictory, or even unachievable demands to make clear the overwhelming power held over the coerced as they (attempt to) meet these demand types; and v) occasional indulgence, which provides motivation for the victim to conform to earn these ‘rewards’ in order to both feel they are maintaining some semblance of self-esteem, and to obtain further rewards in the future.

Similar conditions of psychological captivity are reportedly experienced by victims of trafficking, largely from threats centered around withholding pay, harming the trafficked worker’s family, and contacting the authorities [3, 31, 32]. Other psychological methods of control include deprivation of psychological needs (e.g., no medical care; restricted food and water; limited sleep), and the false creation of debts. Effective psychological control also results from non-violent forms of ill treatment. For example, dire working and living conditions and denial of privacy (e.g., overcrowded living and working conditions), which physically humiliate and degrade victims, and induce physical exhaustion. Personal identification documents, such as passports and driving licences are confiscated, typically immediately upon entering the UK which creates a sense of isolation, reliance on the trafficker, and restricts victims movements from the very offset. Compliance removes trafficked workers free will, creating further isolation and increased dependency on the trafficker [33].

### The Current Study

This research was prompted by the UK draft Modern Slavery Bill, which became law in 2015, and the UK government’s Modern Slavery Strategy and is centered around the public understanding of human trafficking, psychological abuse/coercion with a view to considering how these might impact on the UK government’s desire to prevent trafficking, protect the victims of trafficking, and prosecute the perpetrators of this crime. The very small number of relevant empirical studies on coercion and human trafficking, and Biderman’s framework were used to guide our research approach, and develop our materials.

Controlling crime requires public cooperation, and where the indicators of crime are less than obvious the general population has to be knowledgeable in order to work in partnership with governments and other organisations. A US field study concerning coercion and sex workers has recently highlighted an urgent need to improve US public awareness of trafficking to overcome apathy, naivety and a lack of understanding of the coercive nature of human trafficking [34] a pattern of results which we expect to find replicated in the Midlands of England. Accordingly, the aims of the research were to collect a novel data set recording public perceptions and understanding of psychological coercion and human trafficking in the West Midlands, the objective being to shed some light on current awareness, which may feed into programmes to improve understanding. Despite a dearth of published research, we made a number of tentative hypotheses, as follows;

1. Respondents will have a limited knowledge/understanding of what psychologically coercive behaviour is;
2. Most respondents will have heard of human trafficking, but will have a limited understanding of what human trafficking is;

## Materials and Method

There was little to guide the construction of our materials, other than our research questions/hypotheses, the European Convention on Human Rights Draft Modern Slavery Bill Memorandum, the Modern Slavery Bill Delegated Powers Bill Memorandum (Home Office, 2014), and the US National Crime Justice Association Human Trafficking Task Force [35, 36] were also guided by human trafficking research conducted in the US [33, 34]. The final questionnaire (S1 Appendix) comprised 30 items organized under three section headings, i) About You (3 questions collecting demographic information guided by the UK Office of National Statistics); ii) Coercion (10 questions concerning psychological coercion); and iii) Human Trafficking (17 questions concerning human trafficking), and collected both qualitative and quantitative data variously employing dichotomous and scale questions, along with open-ended invitations across each of the three sections. Once constructed 40 pilot questionnaires were distributed to adults from the general population, with a letter inviting feedback and comment. The clarity of several questions was tightened in response to feedback. In its final form the questionnaire had a good internal consistency with a Cronbach alpha coefficient of .81.

### Sample and Procedure

This research project was conducted in accordance with the British Psychological Society code of ethical conduct. The project in its entirety, including the method of informed consent whereby consent was assumed upon return of the questionnaire, was approved via the University of Wolverhampton Ethics procedure. A total of 850 anonymous questionnaires were distributed between December 2014 and March 2015 to a community area and student sample in the city of Wolverhampton, which is one of the most densely populated local authority areas in England, with a population of 249,470 people living equating to a population density of 3,447 per square kilometre. None of the respondents were known to the research team. Questionnaires were distributed face-to-face, as follows, i) to adults attending community lectures and seminars held at three university campuses in the West Midlands of England, ii) to PG students studying at two Universities in the West Midlands of England, and iii) by opportunity area sampling, approaching adults in Wolverhampton city centre. A covering letter accompanied each questionnaire stating that the purpose of the study was to collect information concerning knowledge of human trafficking. Respondents received no payment for their participation and completed the questionnaire in their own time. Questionnaires were returned either by post, or immediately directly to the researchers in sealed, anonymous envelopes. Written consent was not collected, rather consent was assumed by completion and return of the questionnaire. Of the 850 questionnaires distributed, 682 were completed hence the overall response rate was 80%. Our sample comprised 276 (41%) males and 406 (59%) females, all born in the UK, and resident in West Midlands of England with mean age of 29.9 years (SD = 5.7) ranging from 18 to 64 years (S1 File).

### Knowledge and Perceptions of Psychological Coercion

Respondents were asked to indicate whether they were familiar with the term *psychological coercion* (dichotomous yes/no), and if so where they had read/heard about psychological coercion (from a list of information outlets). Overall 51% (348) of respondents answered yes, they were familiar with the term, and 49% (334) answered no. Of the 348 respondents who had heard of the psychological coercion, the most common source of information (some respondents indicated more than 1 source) was newspapers (47%), followed by online/internet sources (40%), television (30%), social media (26%), and radio (22%).

Although not all respondents were familiar with the term psychological coercion, respondents may have been familiar with some of the types of behaviours deemed as psychologically coercive (see Hooper & Hidalgo, 2006; Kim, 2007). Accordingly, knowledge/perceptions of psychologically coercive behaviours relevant to human trafficking were explored first using one open-ended question inviting respondents to explain what *they* understood by the term ‘psychological coercion’ followed by a series of closed questions giving examples of psychologically coercive behaviours, which respondents were asked to answer using a three point scale, reported in Table 1 below (1 = yes; 2 = not sure, which indicates a neutral response; 3 = no). Questions were posed in this order so that the closed question examples did not ‘prime’ respondent’s replies to the open-ended question.

**Table 1:** Categories (and verbal sub-categories) with exemplar quotes for understanding of psychological coercion (N = 682).

Category	Exemplar Quotes
Imprisonment	When people are locked up so they can’t get out and are only allowed to leave the house for work [99]; Well if people are locked up they can’t leave the house so they are obviously coerced because they have no choice but to stay [421]; If people can’t drive and get locked up in the middle of the country they can’t get away and so they are coerced into doing something they might not otherwise do [112]; Coercion is obviously to do with locking people away or otherwise they would just leave [501]
Verbal threats to withhold money	Telling people they won’t get paid so this keeps them working [17]; People are shouted at and told they won’t get paid, but I don’t know if this coercive–it’s about threatening I think [667]; Telling them that they wont get any money unless they do more work, they must get paid eventually otherwise they wouldn't stay, would they? [519]
Verbal threats to injure	Telling people they won’t get paid so this keeps them working [17]; People are shouted at and told they won’t get paid, but I don’t know if this coercive–it’s about threatening I think [667]; Telling them that they wont get any money unless they do more work, they must get paid eventually otherwise they wouldn't stay, would they? [519]
Verbal threats to report to authorities	Threatening to contact the benefits office [89]; Saying that you will call the tax office coz I bet they don't pay enough tax [100]; Stuff like threatening to call social services, even if they would never do that. It just keeps them in order I guess [77]
Withholding	Making sure they can’t see friends or use their bank account–I bet that is what they do, they stop letting people get to their money? [403]; Removing privileges like going to the cinema and going to restaurants–preventing people from doing stuff and not having any money [397]; Making life really difficult, maybe by not letting them see a doctor [263]

Thematic, summative content analysis [37] was employed to code text responses to the open-ended question into explicit themed categories (themed categories comprise single assertions about the subject/topic in question) which allowed us interpret respondent’s understanding of psychological coercion from the manifest content of replies [3, 38]. Three independent researchers ignorant of the research aims and hypotheses independently coded the qualitative data. Three primary categories (groups of content that share a commonality) emerged from the data, namely i) imprisonment; ii) verbal threats, which we have sub-categorized into three distinct types of verbal threat, namely threats to withhold money, threats to injure and threats to report to the authorities; and iii) withholding (distinct from threats to withhold in that respondents stated that perpetrators actually withheld, rather than threatened to withhold). Inter rater reliability for researcher’s coding of the questionnaires revealed a high degree of agreement between coders for each of the three primary categories, verbal threats, Kappa = .821, *p* = .005; imprisonment, Kappa = .789, *p* = .008; withholding, Kappa = .872, *p* = .001. Table 1 (below) illustrates representative quotes from each of the categories.

The most common type of behaviour believed to be psychologically coercive was physical imprisonment (mentioned by 87% of respondents), followed by verbal threats (mentioned by 42% of respondents) and withholding (mentioned by 37% of respondents). Of the 42% of respondents who believed verbal threats to be psychologically coercive, the most common sub-category was threats to withhold payment (48%), followed by threats to injure (27%) and threats to report to the authorities (20%).

A Friedman test of responses to the six scale questions concerning perceptions/knowledge of types of coercion revealed that participants were significantly more familiar with some coercive behaviours than others (as indicated by a lower mean rank score), χ^2^ (6, *N* = 682) = 180.285, *p* < .001 (see Table 2 for rank order, and population responses). A Kendall coefficient of concordance for these data revealed a significant consensus *W* (5, 682) = .437, *p* = .002.

**Table 2:** Percentage responses and means and SDs, in rank order for knowledge of psychologically coercive behaviours (N = 682).

	Mean	Mean	Yes	Don’t	No
Behaviour	(SD)	Rank	(%)	Know (%)	(%)
Verbally pressure another to commit a crime	1.10(.34)	3.01	626(91.84)	46(6.72)	10(1.53)
Verbally pressure another to behave against their free will	1.32(.67)	3.41	540(79.26)	64(9.43)	78(11.41)
Verbally intimidate another so he/she is no longer able to make decisions	1.35(.68)	3.48	475(69.73)	73(10.75)	134(19.60)
Verbally pressure another to behave against their free will by restricting social contact	1.79(.74)	3.57	374(54.83)	165(24.22)	143(21.04)
Verbally pressure another to behave against their free will by undermining self confidence	1.81(.75)	3.76	298(43.63)	200(29.46)	184(27.03)
Verbally pressure another to behave against their free will by being nice (e.g., buying gifts etc.)	1.99(.74)	3.77	250(36.61)	282(41.44)	150(22.02)

### Knowledge and Perceptions of Human Trafficking

Knowledge/perceptions of human trafficking were explored in the same manner as psychological coercion (open-ended, dichotomous and three point scale questions). Overall, 78% (532) of respondents were familiar with the term ‘human trafficking’, and 22% (150) were not. Means, standard deviations, and percentage responses to questions exploring respondent’s knowledge of human trafficking and the reasons why persons might be trafficked, are displayed in Table 3 (below). Data from 82 respondents was excluded from this analysis because they had not answered *all* eight questions concerning drivers of human trafficking. Analysis of the remaining 615 data sets revealed respondents were more knowledgeable about some of the drivers of trafficking than others (as indicated by a lower mean rank score), χ^2^ (8, *N* = 615) = 669.633, *p* < .001. A Kendall coefficient of concordance for these data revealed a significant consensus *W* (7, 689) = 646.676, *p* < .001.

**Table 3:** Percentage responses and means and SDs in rank order for knowledge of drivers of human trafficking (N = 615).

Reason	Mean	Mean	Yes	Don’t	No
	(SD)	Rank	(%)	Know (%)	(%)
Are adult females trafficked for sexual exploitation	1.35(.62)	3.87	461(67.91)	164(24.03)	57(8.42)
Are people trafficked for organ harvesting	1.38(.66)	3.92	483(70.80)	129(18.91)	70(10.30)
Does trafficking for sexual exploitation exceed trafficking for labour exploitation	1.38(.64)	3.95	469(68.81)	153(22.42)	60(8.84)
Are adult males trafficked for sexual exploitation	1.41(.61)	4.03	421(61.73)	192(28.23)	69(10.13)
Are people trafficked for domestic servitude	1.53(.69)	4.42	304(44.60)	359(52.64)	19(2.78)
Are female children trafficked for sexual exploitation	1.55(.55)	4.62	231(33.90)	353(51.83)	31(4.52)
Are people trafficked to commit crime	1.67(.57)	5.01	178(26.14)	231(33.92)	273(40.01)
Are male children trafficked for sexual exploitation	2.19(.81)	6.17	108(15.82)	155(22.74)	419(61.51)

In response to questions concerning whether human trafficking exists in the UK, whether human trafficking affected respondents lives/whether they believed they came into contact with victims, and whether most victims are ‘rescued’, 68% (465) respondents believed trafficking exists, 22% (149) did not know, and 10% (73) respondents said no. Seventy nine percent (540) of respondents believed that human trafficking did not affect them, 10% (68) did not know, and 11% (75) said yes. Sixty percent of respondents (409) believed that most victims were not rescued or discovered, 29% (198) did not know, and 11% (75) responded yes.

Thematic, summative content analysis was again employed to code text responses to the following three open-ended questions: i) explain in your own words what you think human trafficking is; ii) why do you think people are trafficked; and iii) what do you think makes a person vulnerable to being trafficked. The findings for each question are reported in turn.

### What is Human Trafficking?

Twenty one percent (143) of respondents replied that they did not know what human trafficking was and/or did not answer this question. Analysis of the text responses of the remaining 79% (539) of respondents revealed just two primary categories (groups of content that share a commonality), which were i) movement; and ii) reason. The most commonly stated explanation of human trafficking concerned the movement of people. Overall, 89% (480) of respondents who answered the question believed human trafficking was the ‘smuggling of humans from one country to another’, ‘bringing people over from foreign countries, illegally’, and ‘illegally bringing immigrants into the country’. The words ‘smuggling’, ‘illegal’ and ‘immigrants’ occurred in *all* of the movement responses. The second most commonly stated explanation of human trafficking was centred on the reasons why human trafficking occurred, and so respondents believed it to be a demand behaviour. The majority of respondents, 58% (313), believed people were trafficked was for prostitution, ‘not sure but I think it is prostitution’, ‘smuggling humans for prostitution’, and ‘pushing a person into prostitution’, and 32% (172) of respondents believed people were trafficked for financial gain, ‘using people to make money’, ‘making people work for the minimum wage, and for long hours so that employers benefit’, ‘making people work in certain industries for very low wages’.

### Why Are People Trafficked?

Overall, 15% (102) of respondents did not answer this question. Two primary codes emerged from the responses offered by the remaining 85% (493) respondents, which we have labeled i) trafficker reasons and ii) victim reasons. Trafficker and victim reasons as to why people are trafficked were *all* associated with making/earning money. Forty-two percent (208) of those who answered the question believed that people were trafficked for reasons pertaining to trafficker’s need, that is their need to make money, for example, ‘[traffickers] make them [victims] work, so as to take all their money from them’, ‘to get rich by making other people work for them for very little money’ and ‘to earn money out of other peoples’ misfortune’. Eighty four percent (237) of respondents believed that victims were trafficked to improve their own and/or their families’ lives, for example, ‘Because they are less well off and can earn money for their families’, ‘for money’, ‘to make money’, and ‘as a way of earning lots of money to send for their family’.

### What Makes People Vulnerable to Being Trafficked?

Overall, 22% (150) of respondents either did not answer the question, or replied that they did not know what made people vulnerable to being trafficked. Two primary codes emerged from the responses given by the remaining 532 respondents, which we have labeled, i) person (i.e., individual characteristics); and ii) situation (i.e., situational factors out of the victim’s control). Fifty-seven percent (303) of respondents believed that person characteristics made people vulnerable to becoming a victim, for example, ‘low self-confidence’, ‘being a woman’, ‘being uneducated’, and ‘having a low IQ’. Sixty four percent (340) of respondents believed that situational factors made people vulnerable to becoming a victim, for example, ‘poverty’, ‘no home or money’, ‘no family to care for them’, ‘war’, ‘the economy in their home country’, and ‘having to provide for their family’.

### Sources of Knowledge and Perceptions of Human Trafficking

The most common source of information (some respondents indicated more than 1 source) regarding human trafficking was newspapers (77%), followed by the internet (64%), social media (50%), television (31%), and radio (18%).

## Discussion

Human trafficking is a priority for the UK government. Yet, little is known about public understanding of human trafficking despite the fact that the Modern Slavery Bill is imminently to become law, and the UK government’s human trafficking strategy postulates ‘shared responsibility’ whereby members of the public are charged with assisting the authorities to identify and report instances of this crime. Strategic collaboration of this nature requires communication and shared understanding if it is to be successful. Accordingly, the research reported here is both timely and significant, albeit that we are only able to offer a snapshot of public understanding (from the West Midlands) of human trafficking and a psychological phenomenon that is fundamental to the successful execution of this crime.

### Psychological Coercion

We examined understanding of psychological coercion through the lens of Biderman’s framework of coercion, with reference to the types of nonphysical coercive tactics reportedly experienced by trafficked victims [31]. Our findings are concerning but unsurprising. Despite recent initiatives to raise awareness of human trafficking in the UK, and the international reporting of several high profile cases of trafficking and exploitation of workers, significant numbers of respondents did not fully understand psychological coercion, and what constituted manipulative, nonphysical abuse. We tentatively formulated two hypotheses, around which we have structured our discussion. The first concerned knowledge of psychological coercion. Almost half of our respondents had not heard of the term psychological coercion, however, many were familiar with some psychologically coercive behaviours relevant to human trafficking.

The three most commonly known coercive behaviours (acknowledged by > 65% of respondents) were verbal pressure to commit crime, verbal pressure to behave against free will and verbal intimidation resulting in reduced cognitive functioning and decision-making abilities. This result was unsurprising because for decades these behaviours have been widely reported and conceptualized methods for controlling/coercing intimate partners, in particular. More recently, all have received a significant amount of publicity following convictions for sexual exploitation and other crimes involving coercion in the North East of England, in particular (e.g., BBC, 2014; Daily Mirror, 2013; 2014: Guardian, 2013, 2014). Moreover, ‘controlling or coercive behaviours in an intimate or family relationship’ are specifically mentioned in Clause 76 of the UK Serious Crime Bill, which only achieved Royal Assent in March 2015 following wide public debate and consultation.

The remaining behaviours were known by < 55% of respondents, yet it is these latter coercive behaviours (undermining self-confidence; social control; being nice) that are believed to be among the most prominent in instances of trafficking and other forms of modern slavery[3, 39]particularly during the exploitation stage of the human trafficking process model when individuals are in labour or service circumstances [40]. Effective, but silent (psychological) control during the exploitation stage is of paramount importance to perpetrators so that victims do not draw attention to themselves: outwardly appearing to have free will, despite being systematically exploited, typically for financial gain.

Explanations of psychological coercion revealed fundamental misunderstandings, which we would contend emanate, in part, from the manner in which the UK Government, NGOs and charities often seek to raise awareness of human trafficking. Psychological abuse is not well explained, typically mentioned in brief, and is not highlighted as being key to breaking down victims’ survival responses in order to control them without the use of physical force. Rather, headline-grabbing pictures/images of physical restraint (e.g., hand cuffed victims; victims in prison cells) are often used, which draw attention to the crime, but it could be argued, misinform the public. Hence, that in excess of 65% of respondents erroneously believed psychological coercion to be physical imprisonment is, again, unsurprising. Some respondents did evidence limited understanding that verbal threats, withholding access to services, and denying social contact were also psychologically coercive behaviours. However, by far the most commonly held belief was that coercion was physical in nature, and so the more subtle signs of coercion, fundamental for controlling and manipulating victims, are less likely to be recognized because they appear little known.

### Human Trafficking

Turning to our second hypothesis, which concerned knowledge of human trafficking, we expected that respondents would possess little ‘real’ knowledge or understanding of human trafficking, even if they were aware of the term. This is exactly what we found. The majority of respondents were aware of human trafficking (less than 15% acknowledging that they were unfamiliar with the term), which we suspect is a direct result of the recent publicity associated with the draft Modern Slavery Bill. However, consideration of responses to open-ended questions revealed that most respondents believed that human trafficking was the illegal movement/illegal immigration of people from one country to another, either for prostitution, or work, and that female women were the main victims of traffickers for sexual exploitation purposes.

Respondents were far less aware that men, and female and male children were also trafficked and sexually exploited. Similar perceptions of trafficking being mainly associated with the sex industry have been found in prior studies [21, 22, 41–43] It appears that public understanding of human trafficking directly reflects the representation offered by the UK Government as being an immigration concern underpinned by crime and sexual exploitation, typically affecting women. It has been argued that focusing on sexual exploitation has resulted in other forms of trafficking (such as forced labour and involuntary domestic servitude) being overlooked and that, as a result recognition of human trafficking in these areas is not so readily identified [44, 45]. Our data would indicate this is certainly the case in the Midlands of England.

When asked which groups or individuals were more likely to be vulnerable to becoming victims of human trafficking, financial pressures, an absence of familial support, and the presence of armed conflict where they originally resided, situationally explained human trafficking victimhood. Individual factors (such as a lack of either education or self -esteem, or being female) were, in turn, suggested as causal explanations. The general understanding that human trafficking involved people as ‘slaves’ who are moved from one place to another, against their free will by criminals, did not emerge from our results. Rather, there was clear confusion between human smuggling and human trafficking (where smuggling involves a degree of compliance by those smuggled, certainly in the initial phase, compared to those tricked into human trafficking), which has also emerged in prior research [46–48]. Our respondents either confused immigration and trafficking, or were unaware that they were different, which may lead to trafficked victims being viewed as willing partners in criminality in the eyes of the UK public, compounding their victimization and possibly fueling a degree of ‘serves them right’ indifference. Indeed, almost three-quarters of respondents felt that human trafficking did not affect their lives, despite most believing that trafficking did occur in the UK.

### Limitations

This research only offers a snapshot of public opinion, from one area of England and is subject to the limitations typically associated with such self-report studies, which should be borne in mind when interpreting our findings. Data from a larger sample across a wider area of the UK is now needed, and while self-report questionnaires minimise the effects of interviewer bias and social desirability they are devised by researchers, and so are guided by their research questions and previous theoretical and empirical work. Moreover, they do not allow researchers to gauge the sincerity of the answers provided, nor to explore why some respondents fail to answer questions, for example concerning the drivers of human trafficking. Researchers in this domain should now seek to augment our survey data by conducting semi-structured interviews to develop a more in-depth, qualitative understanding of perceptions and understanding of this crime. In doing so they should fully explore psychological coercion, which is a fundamental aspect of human trafficking. Research should also investigate Perceptions and knowledge of first responders (police, fire fighters, paramedics etc.), who are known to come into contact with potential victims and/or perpetrators of this crime, with a view to developing effective conversational methods for recognizing subtle vulnerability/perpetrator cues at the first point of referral/contact when status is unknown [49].

## Conclusion

The relationship between governmental policy and public understanding and opinion is complex [30]. However, the UK government is unequivocal: public assistance is key to their modern day slavery strategy. Accordingly, investigations of public understanding are necessary. A recent self-report study conducted in the USA argued that public awareness of human trafficking needed to be improved, and to do so Governments and law enforcement agencies were faced with having to overcome apathy, naivety, and a lack of understanding of the psychologically coercive nature of human trafficking [50]. We concur, and suggest that similar efforts may need to be made in the UK. While most of our respondents had knowledge of the term human trafficking, there was a worrying lack of awareness of what it was, how it arose, and what forms it took. This was particularly the case when we examined psychological coercion: understanding was at best patchy, respondents typically believing, erroneously, that indicators were physical in their manifestation.

The public can be one of the most influential interest groups, but only if well informed, supported and motivated towards positive action. Yet in a large city in the Midlands of England many appear largely ignorant of indicators of psychological coercion, and while they believe that trafficking exists (because they have been told) they believe that this crime does not really affect them, and confuse trafficking with illegal immigration, a pattern of results that mirrors those reported by researchers and Government bodies in the US, and elsewhere. Our results indicate that far from the Socratic paradox, whereby the UK Government is aware that little is known, this maybe an example of an unknown, unknown. That is, the UK Government appears less than well informed about public awareness, but nonetheless assumes public knowledge and understanding. Targeted knowledge exchange initiatives that offer information to scaffold the well cited ‘eyes and ears’ expectation is key in the fight against human trafficking, and other forms of modern slavery. Given the range of sources of information relied upon in forming knowledge, a diverse range of targeted communication strategies are necessary (including its causal explanations, indicators, as well as its extent, both in terms of size and diversity) that move away from depicting trafficking as physical imprisonment.

## Supporting Information

S1 Appendix Questionnaire. (DOCX)

S1 File Raw data used in quantitative analysis. (SAV)
