# Supplementary material for: Perceptions of Psychological Coercion and Human Trafficking in the West Midlands of England: Beginning to Know the Unknown
Source: PLoS One. 2016 May 5;11(5):e0153263. doi: 10.1371/journal.pone.0153263 (PMC4858279; doi:10.1371/journal.pone.0153263)
Supplement: S1 Appendix — (DOCX) [file pone.0153263.s001.docx]

**SECTION 1 – ABOUT YOU**

**The section of this questionnaire collects anonymous information about you. For example, where you live and how old you are. This information is important because it allows us to gain a ‘picture’ of who completes our survey. This information is commonly referred to as demographic information.**

1. How old are you (in years)?

_____________________________

1. Are you (please circle):
   1. Male
   2. Female

1. Where do you currently live? ______________________________________________

**SECTION 2 – Psychological Coercion**

**The second section concerns your opinions and understanding of psychological coercion.**

1. Have you heard of the term psychological coercion (please circle)?
   1. Yes
   2. No
2. If you have heard of psychological coercion please indicate **where** you have heard about it (circle all that apply):
   1. Newspaper
   2. Radio
   3. Television
   4. Social media
   5. Internet
   6. Book
   7. Film
   8. Other (please explain) ___________________________________________
3. Please explain what you think psychological coercion means – please use your own words – there is no right or wrong answer. Rather, we would like to know what YOU understand psychological coercion to be:

___________________________________________________________________________________________________________________________________________________________________________________________________________________________________________________________________________________________________________________________________________________________________________________________________________________________________________________________________________________________

___________________________________________________________________________________________________________________________________________________________________________________________________________________________________________________________________________________________________________________________________________________________________________________________________________________________________________________________________________________________

1. Do you think that it is possible to **verbally pressure** another person to behave in a particular manner (against their will) **by undermining their self-confidence**?
   1. Yes
   2. I don’t know
   3. No
2. Do you think that it is possible to **Verbally pressure** another person to behave in a particular manner (against their will) **by restricting social contact** (for example, by limiting their access to friends, family, social media etc.)?
   1. Yes
   2. I don’t know
   3. No
3. Do you think that it is possible to **pressure** another person to behave in a particular manner (against their will) simply **by verbally threatening that person?**
   1. Yes
   2. I don’t know
   3. No
4. Do you think that it is possible to **verbally intimidate another person** to the point where that person is **no longer able to make normal, wise or balanced decisions**?
   1. Yes
   2. I don’t know
   3. No
5. Do you think it is possible to **verbally pressure** another person (against their will) **to commit a crime**?
   1. Yes
   2. I don’t know
   3. No
6. Do you think it is possible to **verbally pressure** a person **to admit to having committed a crime** that they did not actually commit?
   1. Yes
   2. I don’t know
   3. No
7. Do you think that it is possible to verbally **pressure** another person to behave in a particular manner (against their will) **by being nice and treating them well** (for example, by simply spending time, or talking with that person, and/or buying gifts)?
   1. Yes
   2. I don’t know
   3. No

**SECTION 3 - HUMAN TRAFFICKING**

**This section concerns your opinions and understanding of Human Trafficking**

1. Have you ever heard of the term human trafficking (please circle)?
   1. Yes
   2. No
2. If you have heard of human trafficking please indicate **where** you have heard about it (circle all that apply):
   1. Newspaper
   2. Radio
   3. Television
   4. Social media
   5. Internet
   6. Book
   7. Film
   8. Other (please explain) ___________________________________________
3. Please explain what you think human trafficking is (in your own words below):

____________________________________________________________________________________________________________________________________________________________________________________________________________________________________________________________________________________________________________________________________________________________________________________________________________________________________________________________________________

________________________________________________________________________________________________________________________________________________________________________________________________________________________________________________________________________________________________________________________________________________________________________________

1. Do you think that Human Trafficking exists in the UK?
   1. Yes
   2. Don’t know
   3. No
2. Do you think that you come into contact with victims of Human Trafficking?
   1. Yes
   2. Don’t know
   3. No
3. Why do you think that people are trafficked (in general)?

___________________________________________________________________________________________________________________________________________________________________________________________________________________________________________________________________________________________________________________________________________________________________________________________________________________________________________________________________________

_______________________________________________________________________________________________________________________________________________________________________________________

1. Do you think trafficking for sexual exploitation ***exceeds*** that of labour exploitation?
   1. Yes
   2. Don’t know
   3. No
2. Do you think trafficking for ***domestic servitude*** (making a person undertake domestic work, e.g., cleaning, cooking, laundry etc.) exists in the UK?
   1. Yes
   2. Don’t know
   3. No
3. Do you think trafficking for ***organ harvesting*** (a surgical procedure that removes organs or tissues for reuse) exists in the UK?
   1. Yes
   2. Don’t know
   3. No
4. Do you think that human trafficking is an International problem?
   1. Yes
   2. Don’t know
   3. No
5. Do you think ***adult males*** are trafficked for sexual exploitation in the UK?
   1. Yes
   2. Don’t know
   3. No
6. Do you think ***adult females*** are trafficked for sexual exploitation in the UK?
   1. Yes
   2. Don’t know
   3. No
7. Do you think ***male children*** are trafficked for sexual exploitation in the UK?
   1. Yes
   2. Don’t know
   3. No
8. Do you think ***female children*** are trafficked for sexual exploitation in the UK?
   1. Yes
   2. Don’t know
   3. No
9. Do you think people are trafficked in order to commit crime in the UK?
   1. Yes
   2. Don’t know
   3. No
10. What do you think makes a person vulnerable to being trafficked?

_________________________________________________________________________________________________________________________________________________________________________________________________________________________________________________________________________________

___________________________________________________________________________________________

____________________________________________________________________________________________

____________________________________________________________________________________________

1. Do you think that most victims of trafficking are rescued or discovered?
   1. Yes
   2. Don’t know
   3. No

If you would like to make any comments about this questionnaire please write these below:

____________________________________________________________________________________________________________________________________________________________________________________________________________________________________________________________________________________________________________________________________________________________________________
